# Supplementary material for: Genotype–phenotype correlations and novel molecular insights into the DHX30-associated neurodevelopmental disorders
Source: Genome Med. 2021 May 21;13:90. doi: 10.1186/s13073-021-00900-3 (PMC8140440; doi:10.1186/s13073-021-00900-3)
Supplement: Supplementary file 1 — Additional file 1. Supplementary methods. [file 13073_2021_900_MOESM1_ESM.docx]

**Additional information for:**

**Genotype–phenotype correlations, and novel molecular insights into the *DHX30*-associated neurodevelopmental disorders**

**Mannucci *et al*.**

**Additional file 1: Supplementary methods.**

**Expression of 6xHis-SUMO-DHX30** **constructs**

6xHis-SUMO-DHX30 wild-type and mutant constructs were transformed into *E. coli* BL21 (DE3) pLysS pRARE competent cells. Colonies were inoculated into 100 mL of superbroth (SB) medium supplemented with 2% glucose, 0.1% kanamycin and 0.1% chloramphenicol and incubated overnight at 37°C under shaking at 200 rpm. The 100 ml bacterial pre-culture was transferred into 2 L of SB medium and incubated for 3h at 37°C under shaking at 180 rpm until an OD_600_=1 was reached. Then, protein expression was induced by adding 1 mM of IPTG and cells were cultured overnight at 14°C under shaking at 180 rpm.

Cells were collected by centrifugation and cell pellets were resuspended in 50 mL of lysis buffer (20 mM HEPES pH 7.5, 500 mM NaCl, 5 mM MgCl_2_, 0.01% NP-40, 10% glycerol, 5 mM β-mercaptoethanol, 0.1 mM AEBSF, 0.5 mg/L leupeptin/pepstatin A, 2 mg/L aprotinin). Solubilized samples were additionally sonified with the Brandson 250 sonifier. Insoluble cell debris was removed by ultracentrifugation with a 45Ti Beckman rotor at 25 000 rpm for 1h at 4°C.

**Protein purification.** Cell lysates were incubated for 2h at 4°C on Ni-NTA beads (Qiagen, #30210) previously equilibrated with lysis buffer. Then, beads were washed three times with 50 times bed volume of washing buffer (20 mM HEPES pH 7.5, 500 mM NaCl, 5 mM MgCl_2_, 0.01% NP-40, 10% glycerol, 40 mM Imidazole, 5 mM β-mercaptoethanol, 0.1 mM AEBSF, 0.5 mg/L leupeptin/pepstatin A, 2 mg/L aprotinin). Bound proteins were eluted with 8 times bed volume of elution buffer (20 mM HEPES pH 7.5, 500 mM NaCl, 5 mM MgCl_2_, 10% glycerol, 300 mM imidazole, 5 mM β-mercaptoethanol,). The elution fractions were subsequently analyzed for the presence of the target protein by SDS-PAGE and Coomassie blue staining. Afterwards the fractions of interest were pooled and sample was concentrated using concentrators (Vivaspin™ 500, MWCO 10 000). 2 - 5 mg of the concentrated sample was further purified by size exclusion chromatography (Superdex 75) with the Äkta™ purifier system (GE Healthcare). Fractions were analyzed for the presence of the target protein by SDS-PAGE and Coomassie staining. Samples were quantified from the Coomassie stained gel using the ImageJ software.

***In vitro* synthesis of RNA molecules.** To test the RNA unwinding activity of DHX30, a ^32^P-Labeled RNA duplex was synthesized using the T7 RNA polymerase from a linearized DNA template designed by (Tseng-Rogenski and Chang, 2004). The *in vitro* transcription reaction mix was prepared as follows: 1X transcription buffer (40 mM Tris-HCl pH 7.9, 1 mM Spermidine, 26 mM MgCl2, 0,01% Triton X, 5mM DTT), NTPs (GTP 8 mM, ATP 5 mM, CTP 5 mM, UTP 2 mM, 50 µCi of ^32^P-UTP), 3 µM DNA template, 3 µM top strand primer (5’-TAATACGACTCACTATAG-3’), 7 U of T7 RNA polymerase. Transcription reactions were incubated for 2h at 37° C. RNA was precipitated by adding 0.1 volumes of 3M NaAc (pH 5.5) and 3 volumes of ethanol for 30 min at -20°C. Then, samples were centrifuged at 13 000 g, 30 min at 4°C. RNA pellets were rinsed with 70 % ethanol and resuspended in H_2_O.

RNA samples were mixed with 2X denaturing RNA loading dye (1X Tris-Borate-EDTA pH 8.3, 95% formamide, 0.1% bromophenol blue, 0.1% xylene cyanol FF) and analyzed on 8% UREA-PAGE in 1X TBE. Radioactive signals were detected by autoradiography and the RNA band was excised from the gel.

The RNA product was extracted from the gel in RNA extraction buffer (200 mM Tris-Hcl pH 7.0, 0.1% SDS, 1 mM EDTA). Subsequently, RNA was precipitated as described above and the RNA pellets were resuspended in 100 mM KCl.

To promote the formation of the RNA duplex, the RNA sample was boiled at 95°C for 5 min and cooled down over 2h. The sample was mixed with 2X non-denaturing loading dye (1X TBE, 20% glycerol, 0.1% bromophenol blue, 0.1% xylene cyanol FF) and separated on 8% native PAGE. The RNA duplex was excised from the gel and purified as described before. The RNA concentration was determined by measuring absorbance at 260 nm.

**RNA unwinding assay**

The helicase activity was measured in 20 µl of reaction mixture containing 0.13 pmol of purified protein (=20 ng of full length protein), 25 fmol radioactively labeled RNA duplex, 17 mM HEPES-KOH pH 7.5, 150 mM NaCl, 1 mM MgCl_2_, 2 mM DTT, 1 mM spermidine, 0.3% PEG8000, 5% glycerol, 150 mM KCl, 20 units of RNasin™ Plus (Promega), 1 mM ATP. The mixture was incubated for 1h at 37°C and subsequently mixed with 2X non-denaturing loading dye and subjected to gel electrophoresis through non-denaturing 8% PAGE (19:1) in 0.5X TBE at 4°C. Reaction products were visualized by autoradiography.
