# Supplementary material for: Genotype–phenotype correlations and novel molecular insights into the DHX30-associated neurodevelopmental disorders
Source: Genome Med. 2021 May 21;13:90. doi: 10.1186/s13073-021-00900-3 (PMC8140440; doi:10.1186/s13073-021-00900-3)
Supplement: Supplementary file 3 — Additional file 3: Figure S1. Identified missense variants affect highly conserved amino acids. [file 13073_2021_900_MOESM3_ESM.docx]

**Additional information for:**

**Genotype–phenotype correlations, and novel molecular insights into the *DHX30*-associated neurodevelopmental disorders**

**Mannucci *et al*.**

**Additional file 3**

**
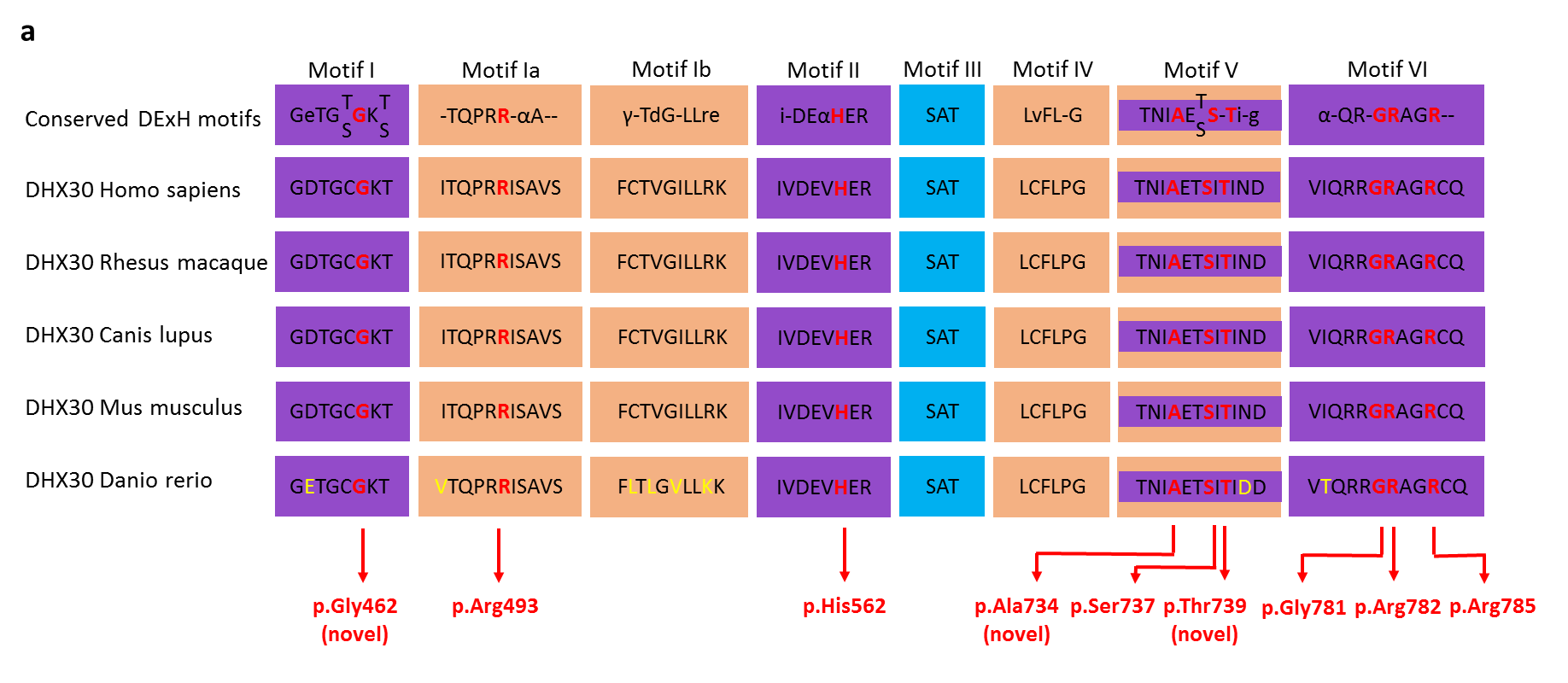
**

**
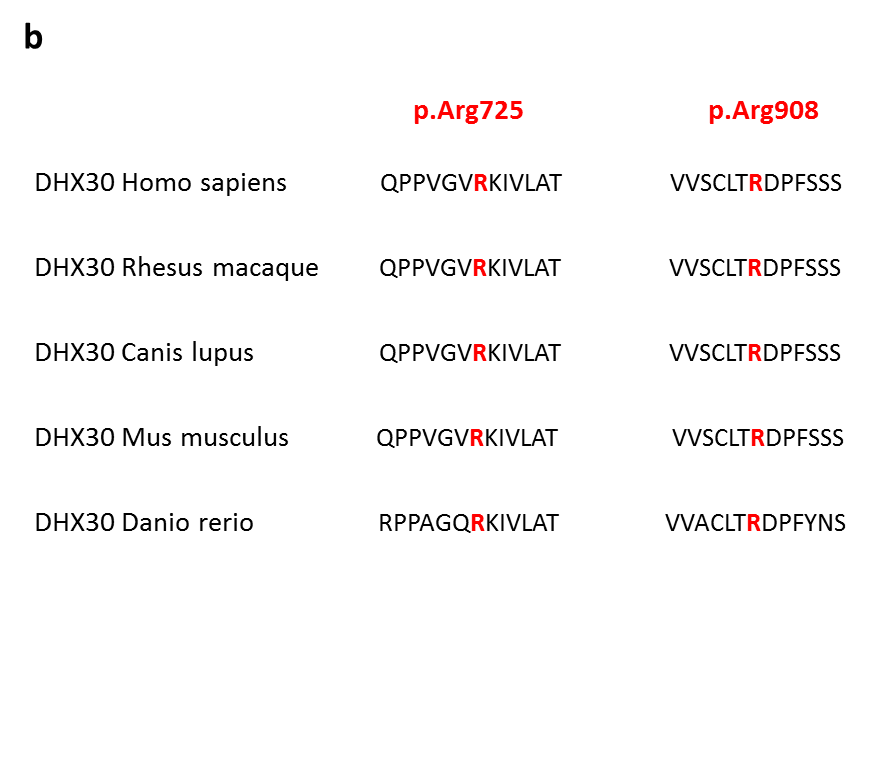
**

**Fig. S1. Identified missense variants affect highly conserved amino acids.** (**a**) Evolutionary conservation of the missense variants within motifs of the helicase core region. The position of the missense variants identified are shown in red. Amino acid residues affecting novel missense variants are noted in brackets. Non conserved amino acids are shown in yellow. Nucleotide-interacting motifs (I, II and VI) are shown in purple, nucleic acid-binding motifs (Ia, Ib and IV) in orange, motif V, which binds nucleic acid and interacts with nucleotides, in purple and orange, and motif III, which couples ATP hydrolysis to RNA unwinding, in blue (as previously described by Lessel et al., 2017). (**b**) Evolutionary conservation of the missense variants p.(Arg725His) and p.(Arg908Gln) not located within motifs of the helicase core region. The positions of the missense variants are shown in red. Note that the affected amino acids are evolutionary highly conserved from humans to zebrafish.
