# Supplementary material for: Genotype–phenotype correlations and novel molecular insights into the DHX30-associated neurodevelopmental disorders
Source: Genome Med. 2021 May 21;13:90. doi: 10.1186/s13073-021-00900-3 (PMC8140440; doi:10.1186/s13073-021-00900-3)
Supplement: Supplementary file 4 — Additional file 4: Figure S2. De novo mosaicism in individual 6. [file 13073_2021_900_MOESM4_ESM.docx]

**Additional information for:**

**Genotype–phenotype correlations, and novel molecular insights into the *DHX30*-associated neurodevelopmental disorders**

**Mannucci *et al*.**

**Additional file 4**


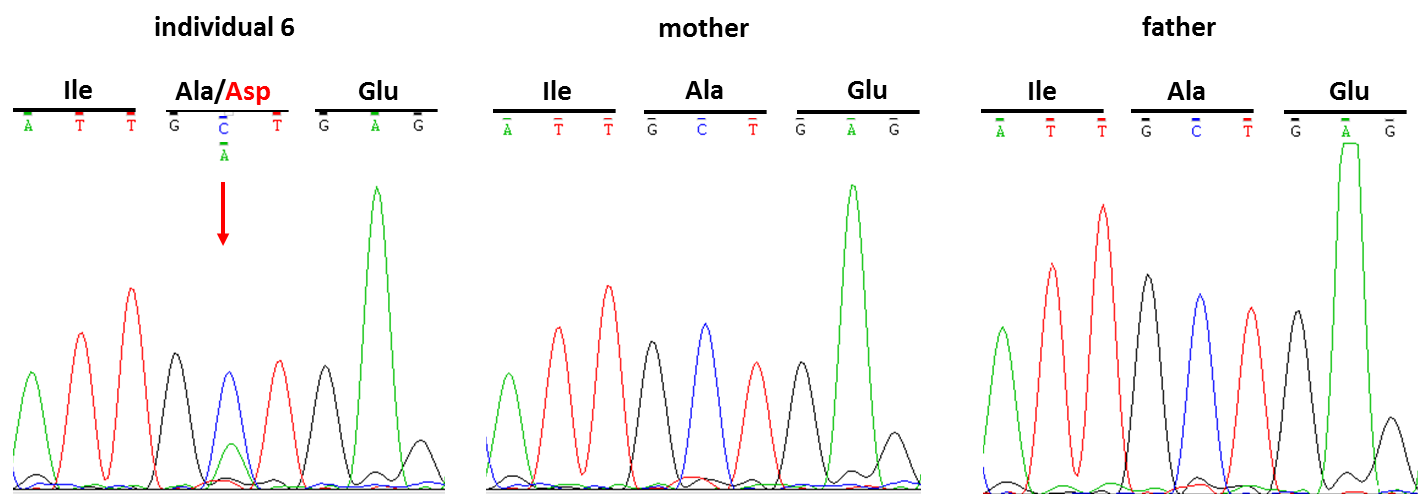


**Fig. S2. *De novo* mosaicism in individual 6.** Sanger sequence electropherograms of parts of *DHX30* after PCR amplification of genomic DNA of the affected individual 6 and her parents, confirming *de novo* mosaicism. The amino acid translation is shown in the three-letter code above the DNA sequence. The red arrow indicates the variant at c.2201C>A, p.(Ala734Asp) present only in the DNA sample of the affected individual.
