# Supplementary material for: Genotype–phenotype correlations and novel molecular insights into the DHX30-associated neurodevelopmental disorders
Source: Genome Med. 2021 May 21;13:90. doi: 10.1186/s13073-021-00900-3 (PMC8140440; doi:10.1186/s13073-021-00900-3)
Supplement: Supplementary file 5 — Additional file 5: Figure S3. Whole gene deletion in individual 24. [file 13073_2021_900_MOESM5_ESM.docx]

**Additional information for:**

**Genotype–phenotype correlations, and novel molecular insights into the *DHX30*-associated neurodevelopmental disorders**

**Mannucci *et al*.**

**Additional file 5**

**
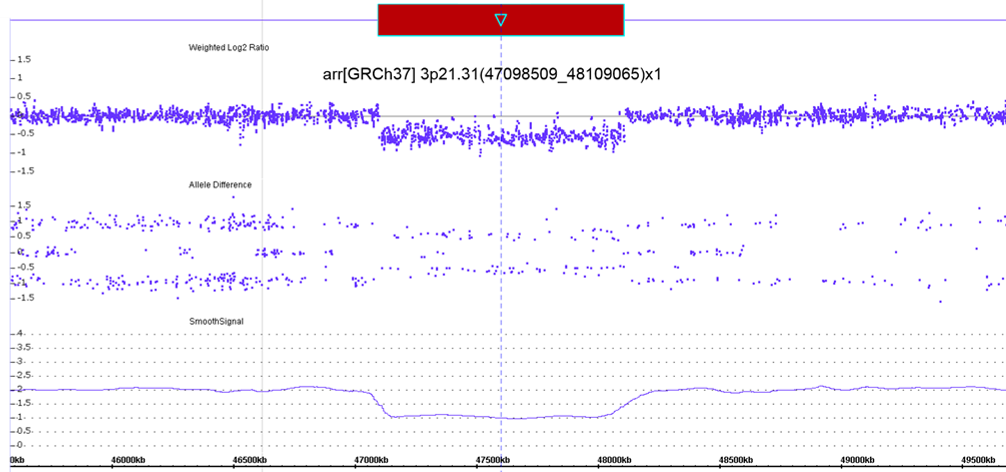
**

**Fig. S3. Whole gene deletion in individual 24.** Adapted from Chromosome Analysis Suite 3.3 (ChAS 3.3) showing loss of oligonucleotide probes at 3p21.31. Each dot represents one single nucleotide polymorphism that are distributed on the x‐axis which shows the genomic positions.
