# Supplementary material for: Genotype–phenotype correlations and novel molecular insights into the DHX30-associated neurodevelopmental disorders
Source: Genome Med. 2021 May 21;13:90. doi: 10.1186/s13073-021-00900-3 (PMC8140440; doi:10.1186/s13073-021-00900-3)
Supplement: Supplementary file 6 — Additional file 6. Clinical reports of here presented individuals. [file 13073_2021_900_MOESM6_ESM.docx]

**Additional information for:**

**Genotype–phenotype correlations, and novel molecular insights into the *DHX30*-associated neurodevelopmental disorders**

**Mannucci *et al*.**

**Additional file 6: Clinical reports:**

Individual 1 is a 9-year-old female, the first child of non-consanguineous parents of Caucasian European ancestry. Her older maternal half-brother, mother and maternal grandmother were regarded to have familial hypermobility / Ehlers- Danlos syndrome. Otherwise family history was inconspicuous. Pregnancy and birth were uncomplicated. At 6 months of age her elbow joint was dislocated without any trauma. She could roll over spontaneously at 9 months of age. At 18 months she was able to sit on her knees with malrotated abducted lower legs. At 2 years of age she could stand with support. She was hypermobile and could fall asleep with her head between legs. She was referred for a genetic evaluation at 3 years of age due to muscular hypotonia, joint hypermobility, feeding difficulties, delayed milestones of development and an occipitofrontal head circumference (OFC) of 45 cm (-4.0SD), i.e. -3 cm below 2.5 percentile. Clinically she was suspicious of absences. However, EEG gave normal results and brain MRI revealed somewhat cerebral atrophy, increased subarachnoidal space and marked ventricles. At clinical investigation at 3.5 years of age she had an OFC of 45.5 cm (-3.9 SD), she had a marked muscular hypotonia, joint hypermobility, was non-verbal, had a convergent strabismus of 20 degrees, and a tendency to stand on her toes with support of parent. She started using a walker at the 5 6/12 years of age. At the last clinical examination at 6 years of age she had an OFC of 45.5 cm (-4.6 SD), was non-verbal, could stand alone and use a walker. Antiepileptics, Sodium valproic acid, had some effect on sleep and daily function. Conventional chromosome analysis of lymphocytes, Array Comparative Genomic Hybridization (array-CGH), Multiplex Ligation-dependent Probe Amplification (MLPA) for *ATRX*, Ehlers-Danlos type III&IV, Ehlers-Danlos type VI and Prader-Willi/Angelman, next-generation sequencing (NGS) panel for Ehlers-Danlos syndrome (9 genes), all gave normal results. Trio whole exome sequencing (trio-WES) with DNA samples of both unaffected parents and the proband, revealed a *de novo* heterozygous variant c.1385G>A, p.(Gly462Glu) in *DHX30* (NM_138615.2).

Individual 2 is a 4-year-old male, the second of two children of unaffected, non-consanguineous parents of African American ancestry. Family history was non-contributory. He was born at 38 6/7 weeks gestation by repeat cesarean to a 36 year old G2P1-2 mother weighing 2940gm (<1.5SD) with a length of 45.7cm (<2SD) and OFC of 34.3cm (<0.3SD). The pregnancy was complicated by maternal anemia. He had mild feeding issues after birth and was in the NICU for a total of 5 days and was then discharged home. He was born with post-axial polydactyly of both hands and one foot. All of the additional digits had a bony component, and were surgically removed at 3-months. Psychomotor development was severely delayed. Physical exam at 4 years old identified an acquired microcephaly, with an OFC of 47cm (<2.5SD). His length was 93.5cm (<2SD) and his weight was 12.2kg (<2.5SD). He had no significantly dysmorphic features. At 4 years old, he was able to babble, but had no specific words. He followed a few simple commands. He had a history of aspiration and required thickened liquids and mechanically soft foods. He could use a fork and a spoon, but would often over-stuff his mouth. He learned to walk at 2 ½ years, but did not yet run and exhibited ataxia and toe-walking. He could assist in dressing, but was not yet toilet trained. He had a normal brain MRI at 21 months of age. He has had no seizures. A chromosomal microarray at 19 months of age identified a maternally inherited 15q13 duplication which was considered to be a variant of unknown significance. He also had a normal Fragile X test at that time. WES at 3 years of age revealed a heterozygous variant c.1478G>A, p.(Arg493His) in *DHX30* (NM_138615.2).

Individual 3 is the first daughter of unaffected and non-consanguineous couple with unremarkable family history. She was born following a normal pregnancy with a birth weight of 3231 grams. There were no significant neonatal problems reported; and her parents feel that her early development was within normal limits for the first 6 months. Following this, she started displaying signs of developmental delay and muscular hypotonia, which has been understood as regression. She has also had a history of bronchomalacia, frequent upper respiratory tract infections and vomiting, and very poor eye contact. Her growth pattern was age appropriate. She was able to sit up at 1year, crawled at 20 months, and learned to walk with 54 months. She presented with stereotypic hand movements; and due to these features, the diagnosis of Rett Syndrome was considered at age 2 years. At the clinical examination at the age of 6 and a half years she was noted to have some distinctive features, such as thick hair, thick vermillion of lower and upper lips, deep set eyes. She also had generalized hypotonia and brisk reflexes on her lower limbs. She was making very slow developmental progresses, and never actually developed expressive speech. She had some swallowing difficulties, problems with chewing and history of severe regurgitation. Neuro-metabolic work-up has been within normal limits. A brain MRI revealed abnormal white matter changes in the posterior horns of both lateral ventricles. She had several EEG's, with no specific features. She did not suffer from epilepsy. Presently at 25 years, she no major medical problems. She has a friendly personality and is always smiling. She does not speak; hence she communicates using an iPad (iGaze), which is working very well for her. She still has some difficulties swallowing and is dependent for feeding. Her gait is unsteady. Her mother suspects a propensity for ear infections (she had several pseudomonas infections). Moreover, she was investigated for Postural tachycardia syndrome (PoTS), as she was suffering from tacchycardia and sweets episodes; but a cardiac assessment gave normal results. Over the years, conventional chromosome analysis of lymphocytes, array-CGH, genetic testing for Rett syndrome, SMA, Pitt-Hopkins syndrome and myotonic dystrophy, chromosome 15 deletion for Prader-Willi Syndrome, Fragile-X testing, all gave normal results. Without a diagnosis, in 2013 she was recruited to the DDD study. WES revealed a heterozygous variant c.1685A>G, p.(His562Arg) in *DHX30* (NM_138615.2).

Individual 4 was a boy, the third child of unaffected, consanguineous parents born at 40 weeks of gestation, with a birth weight of 3220 grams (-0.9SD). Regarding family history, the child's father had one paternal and one maternal uncle with epilepsy. He was admitted to pediatric resuscitation unit because of seizures and myoclonia of the left hemibody, with consciousness disorders and a hypertonia of the left hemibody. The electroencephalogram (EEG) showed a slow sleep path slightly pathological. Then, there were repeated hospital admissions. Two weeks later, the clones in the lower left limb resolved. But at the age of 4 months, he again presented myoclonia. The EEG showed centrotemporal focal spots of spikes. The brain MRI revealed a diffusion restriction affecting the sub-cortical white substance of the left on the pre central convolution and the posterior part of the upper frontal convolution; associated with meningeal lesions evoking a localized inflammatory process. At the clinical examination at 5 months, he showed no eye contact, an axial hypotonia, with agitation between the crises. The EEG showed a very slow track with abundant diffuse wave spikes and a right rhythmic delta burst, and also a diffuse wave peaks, which predominate at the right and left centro-temporal regions. He was admitted to hospital for gastroenteritis at 6 months. At 10 months, he was admitted to the pediatric emergency room for increased seizures after a vaccination one week earlier, with onset of more frequent contact ruptures and cloning of the right upper limb for prolonged periods. Feeding difficulties were observed over the following days. Three weeks after, he was again hospitalized with decreased movements of the eyeballs; a much altered contact, a major axial hypotonia, and repeated opisthotonos attacks as well as an oral facial dyskinesia and myoclonias of the left hand which disappeared completely in deep sleep and increased at stimulation. The EEG showed a hypsarrhythmia traversed from spikes to type of repetitive paroxysms predominant on the right. He deceased at the age of 11 months. Standard metabolic screening gave normal results. Conventional chromosome analysis of lymphocytes, array-CGH, genetic testing of mtDNA for MELAS, MERRF and NARP, for eIF2B-related disorders, as well as analyses of *SCN1A* and GABRG2 gave normal results. The trio-WES, with DNA samples of both unaffected parents and the proband, revealed a homozygous variant c. 2174G>A, p.(Arg725His) in *DHX30* (NM_138615.2). No other candidate variant was identified.

Individual 5 is an 11-year-old female of unaffected and non-consanguineous couple with unremarkable family history. She has 2 healthy brothers. She was born after an uncomplicated pregnancy and a normal delivery at 41 weeks and 3 days of gestation with a birth weight of 2930 g (-1.7 SD) and a birth height of 48.5 cm (-1 SD) and a OFC of 33.5 cm (-1.8 SD). Development was apparently normal up to 4 months with a smile response and the start of voluntary grasping before 3 months. Parents describe deterioration of her condition at around 4 months following an episode of chickenpox infection. A brain MRI at 11 months revealed cortical and subcortical atrophy, and delayed myelination. She later developed hand stereotypies of the middle line and autistic features.at the last clinical examination at the age of 11 years we saw a girl with some dysmorphic features including a short forehead, likely related to her microcephaly, arched eyebrows, a slight synophris, upslanted palpebral fissures, anteverted nares, short columella, and smooth philtrum. There is a delay in the fall of the lacteal teeth. Further, she has micrognathia and high palate. Skin is thin with an apparent venous network and a slight hypertrichosis was noticed. She did not yet acquire autonomous walking, and is wheelchair-bound using it by advancing her arms and then both legs together. Fine motor skills are mostly absent. She can speak only 4 words: “mom” “dad” “yes” and “no”, but can communicate a few words in sign language. She had repeated pulmonary aspirations and prefers grinded food. She has a general hypotonia, significant hyperlaxity with genu recurvatum of the knees and pes valgus. There is no pyramidal or extrapyramidal syndrome. She developed postnatal microcephaly with -5 SD, a severe delay in weight (-3 SD) and height (-5 SD) gain. She developed strabismus, further ophtalmological and auditive examination gave normal results. Chromatography analysis revealed ammonemia and AICAR-SAICAR accumulation. Array-CGH and Fragile X analysis gave normal results. Trio-WES with DNA samples of both unaffected parents and the proband, revealed a *de novo* heterozygous variant c.2201C>A, p.(Ala734Asp) in *DHX30* (NM_138615.2) which was confirmed by Sanger sequencing.

Individual 6 is a 15-year-old female, the first of three children of healthy non-consanguineous parents of Aramean descent. The family history was unremarkable; in particular no developmental disorders have been reported and both younger siblings are healthy. The girl was born after an uneventful pregnancy at term with normal birth measurements (weight 3290 g (-0.5 SD), length 54 cm (1.0 SD), OFC 35 cm (0 SD)). Soon after birth she was admitted to the ICU because of cyanotic events and hypotonia; she was discharged after a few days. Hypotonia persisted, and she developed feeding difficulties and failure to thrive. A situation resembling Acute Life- Threatening Event (ALTE) occurred at the age of 3 months, and a unique febrile seizure followed at the age of 6 months. Motor and speech development were severely delayed; she started to walk without support at 2 years and 8 month, but gait was unstable and she fell down many times over the next years. She spoke first at 2 years and the language development was not appropriate for her age. Receptive language was much better. During toddlerhood, feeding difficulties switched to hyperphagia, she had no feeling of satiety. Thus, she gained weight and became obese (30.9 kg, +1.4 SD) at the age of 11 years. In addition she developed aggressive behavior with autistic features and sleep disturbances. She attended a school for children with special needs. Analysis of blood and urine showed slight and unspecific elevation of glycosaminoglycan’s, but extensive metabolic work up did not detect any additional abnormal results. ECG, EEG, echocardiography, abdominal ultrasound, and brain MRI were unremarkable. Hearing test and eye examination gave normal results. At the last examination at the 15 years of age, we saw a friendly and shy young woman who answered simple questions with simple sentences. She loved to play football although her gait was still ataxic. She was able to read single letters and write single words. She was well- integrated in her family as well as at school; her parents reported that she helps with household chores. She has not shown aggressive and autistic behaviors anymore, but developed an anxiety disorder. Her height was 151.5 cm (-1.1 SD), weight was 49 kg (+0.1 SD) and OFC was microcephalic with 52 cm (-1.6 SD). Conventional chromosome analysis of lymphocytes gave normal results. Trio-WES with DNA samples of both unaffected parents and the proband, revealed mosaicism for a *de novo* heterozygous variant c.2201C>A:p.(Ala734Asp) in *DHX30* (NM_138615.2), identified in 36 out of 174 reads (21%). The *de novo* occurrence of the mosaicism was confirmed by Sanger sequencing.

Individual 7 is a 33-year old male who was born by normal delivery at term following an uneventful pregnancy. The family history was negative for similarly affected individuals and his non-consanguineous parents were of European and Hispanic ethnicity. He developed normally until 8 months of age, when he started to regress. He has had severe developmental delays. He started to walk at age 12 years, but has not developed speech. Dystonia and chorea commenced at 10 years of age and he has also developed tics. At 33 years of age, he used a wheelchair for ambulation and he was non-verbal, although he could communicate with sounds. He did not consistently obey commands and his verbal comprehension was difficult to assess, but he was interactive and affectionate with his family. He has had dysphagia and required a g-tube for liquids, but can eat solids by mouth. Other medical problems have included gait ataxia, hypotonia, cortical blindness, sleep disturbances, constipation and urinary incontinence. He suffered a single febrile seizure at two years of age. On examination at 28 years of age, height was 162.6 cm (3^rd^ centile) and weight was 38.56 kg (<1^st^ centile). His head circumference was 53 cm (8^th^ centile). He demonstrated dystonia and tics with writhing movements of his arms. There were prominent supraorbital ridges, mildly overfolded right ear helix, a narrow nasal bridge and a small and narrow jaw. He had left esotropia and leukocoria. He had wrist and finger hypermobility and a thoracic scoliosis concave to the left. His 4th and 5th toes were small and curled. Neurological examination showed increased tone in all limbs, with brisk reflexes. An MRI of the brain showed mild diffuse prominence of the cerebrospinal fluid space and mild asymmetric prominence of right superior ophthalmic vein. Metabolic investigations, including ammonia, carnitine levels and acylcarnitine profile, serum amino acids, urine organic acids, testing for congenital disorders of glycosylation, copper, ceruloplasmin and pantothenic acid levels were non-diagnostic. Conventional chromosome analysis of lymphocytes, fragile X syndrome testing and single nucleotide polymorphism (SNP) array gave normal results. Methylation studies for Prader-Willi syndrome/Angelman syndrome were negative and mitochondrial testing was also unrevealing. WES, performed as a duo with his mother, identified a heterozygous variant c.2215A>G, p.(Thr739Ala) in *DHX30* (NM_138615.2). His father was unavailable for testing.

Individuals 8 and 9 are half-sisters born to the same mother but have different fathers. The mother has no physical health problems and does not have any history of developmental delay or intellectual disability. She has a history of anxiety, depression and substance abuse. Her examination did not reveal any evidence of abnormal skin pigmentation or body asymmetry that would indicate a possible mosaic disorder. There is no other relevant family history.

Individual 8 was born by spontaneous vaginal delivery at term. She was able to sit with support from the age of 2 years. She achieved independent walking at the age of 10 years. She had limited vocalization and had 10-15 words by the age of 5 years. Currently at the age of 14 years, she can speak only single words. At the age of 12 years, she started having short-lasting and self-correcting vacant episodes that were associated with head dropping, eye rolling and loss of body tone. She had one particularly severe episode where she went absent and then her left arm lost power. There were no observed focal seizures. She was commenced on sodium valproate however this did not recur and she has since been weaned from this medication successfully. She continues to have absent episodes but these are thought to be non-epileptic in nature as they are short-lived and she can be easily roused from them. She has a round face, brachycephaly, large ears, bilateral epicanthic folds, open mouthed expression, everted lips and micrognathia. She has cold plethoric hands. Her growth parameters at 2 years 4 months were: height 87.5cm (-0.25 SD), weight 9.2kg (-3.33 SD) and OFC 46.5cm (-0.95 SD). Her OFC at 7 years 10 months was 48.3cm (-2.65 SD). An EEG and cranial MRI were both unremarkable. She also had normal CSF cell count and biochemistry, VLCFA, CSF amino acids and TORCH screen.

Individual 9 is the younger half-sister of individual 8, born by spontaneous vaginal delivery at term. She did not require resuscitation but was admitted to SCBU for 5 days due to poor feeding and hypernatraemic dehydration. There were no obvious neonatal seizures however she is noted to be jittery, possibly secondary to neonatal opiate withdrawal. She attained social smile on time. At 9 months of age she was noted to be vocalizing minimally. She was noticed to be poorly fixing but an ophthalmological examination did not identify any abnormalities. At 2years 11months, she had no speech, was sitting with support but not independently. At last clinical review, age 6 years, she has a standing frame but her mobility mainly consists of rolling around. She is able to get into a crawling position and sit herself up for a very brief period. Her speech remains unintelligible as she shouts and has no obvious words. She had experienced two chest infections requiring admission to hospital. At this time she was noted to have short-lasting vacant episodes. It is unclear clinically if these episodes could be behavioural although seizures remain a possibility. She has not required any anti-epileptic medication. She has severe reflux requiring treatment with omeprazole and domperidone. She has plagiocephaly, a bifid uvula, high palate and single left palmar crease. Her feet and external genitalia were normal. She continued to have poor visual fixation and was also noted to have cold peripheries, 2-3 mild syndactyly and small nails on the 5th toe bilaterally. Her growth parameters at 9 months were: height 66.4cm (-1.55 SD), weight 6.25kg (-2.30 SD), OFC 41cm (-2.11 SD). At 2 years 11months, her height was 76.9cm (-4.36 SD), weight was 7.65kg (-6.73 SD) and OFC was 44cm (-2.83 SD). An awake and sleep EEG were both normal but cranial MRI showed partial agenesis of the corpus callosum with possible frontal atrophy. Urine organic and amino acids revealed no abnormality. She was thought to have the same condition as her elder half-sister.

In both girls, Angelman testing (FISH 15q and 15q methylation studies), 7-dehydrocholesterol and white cell enzymes gave normal results. Chromosomal microarray analysis in individual 8 revealed no abnormalities. In individual 9, chromosomal microarray revealed a possible duplication at 22q12.2:q12.3(31592382-32217094). This gain encompasses *RNF185*, *LIMK2*, *PIK3IP1*, *PATZ1*, *DRG1*, *EIF4ENIF1*, *SFI1*, *PISD*, *PRR14L* and *DEPDC5* genes. However, as both the affected girls were thought to have the same condition, this gain was thought to be co-incidental. WES was performed in individual 8 as part of the Deciphering Developmental Disorders (DDD) study and identified heterozygous variants in *DHX30* (chr3:g.47889727C>T, ENST00000445061 c.2344C>T, p.Arg782Trp), *RHOBTB2* (chr8:g.22864764C>T, ENST00000519685 c.1072C>T, p.Arg358Ter) and *KAT6A* (chr8:g.41798484C>G, ENST00000396930 c.2915G>C, p.Arg972Pro).

Variants in *RHOBTB2* have been associated to an autosomal dominant epileptic encephalopathy (OMIM #618004) however all pathogenic variants are missense ones, and a gain-of-function mechanism has been postulated. The p.Arg358Ter variant in individual 8 inserts a premature stop codon in exon 7 out of 12, which is likely to undergo nonsense-mediated decay. This variant does appear in healthy controls at low frequency (0.000008 mean allele frequency). There are also seven copy number losses encompassing *RHOBTB2* on the database of genomic variants which lends further support to loss-of-function not being the pathogenic mechanism. Thus, this is thought to be a low frequency benign variant. Pathogenic variants in *KAT6A* cause autosomal dominant mental retardation (OMIM #616268) with nearly all described variants being frameshift or nonsense. The missense variant in individual 8 is predicted to be benign by in silico tools and is also seen infrequently in healthy controls (mean allele frequency 0.00001) hence it is believed to be benign. Targeted Sanger sequencing for the c.2344C>T *DHX30* variant identified it to be present in the affected half-sister (individual 9). However, targeted Sanger sequencing did not show presence of this variant in the DNA samples extracted from either peripheral blood or saliva of the mother of the two children. Hence we suspect this is a case of gonadal mosaicism given both girls have separate fathers.

Individual 10 is a 3 years old female who was the product of an uncomplicated pregnancy. She was delivered to an unaffected Caucasian couple at 39 weeks of gestation with a birth weight of 3110 grams (-0.6 SD) and length of 48 cm (-1.4 SD) and a OFC of 33.5 cm (-0.9 SD). APGARs were 8 and 9. She was noted to have a heart murmur and an Echocardiogram at 3 days of life showed a small anterior/apical ventricular Septal Defect. The Newborn screening was normal. She had a healthy sister and a healthy paternal half-brother. The maternal half- uncle had cerebral palsy and her paternal aunt had severe epilepsy and intellectual disability. She was referred to the clinical genetics because of developmental delay as well as feeding problems as early as 7 months of age. Hypotonia, mild facial dysmorphism and generalized joint hypermobility were noted. She had rotatory nystagmus which improved in time. The brain MRI at 13 months of age showed delayed myelinization (6-8 months) and delayed corpus callosum development with left hippocampal atrophy. At 3 years old age, she has two words and is able to sit up, roll and is starting to crawl. She is also pulling herself up but is not walking. She has two words and is starting to use a communication device. She can finger-feed herself. Her weight is just below the 5th percentile and her length is at the 7th percentile. Chromosomal microarray analysis, DNA testing for Prader Willi Syndrome and Spinal Muscular Atrophy gave normal results. Trio-WES with DNA samples of both unaffected parents and the proband, revealed a *de novo* heterozygous variant c.2344C>T, p.(Arg782Trp) in *DHX30* (NM_138615.2).

Individual 11 is a 7-year-old female, product of an uncomplicated pregnancy following a natural conception. Family history is negative for a similar phenotype; the patient’s younger sister is unaffected, and younger brother was diagnosed with high functioning autism spectrum disorder; her parents are healthy of Caucasian descent, consanguinity was denied. She was born by a normal spontaneous vaginal delivery with a birth weight of 2.67 kg. due to poor sucking she required syringe feeding in the first few days of life. She has been followed by Pediatric Neurology, Pediatric Gastroenterology, and Medical Genetics for intractable complex partial epilepsy; profound global developmental delay and intellectual disability (non-verbal); athetoid cerebral palsy (wheelchair dependent); and failure to thrive (G-tube dependent). Brain MRI with spectroscopy performed at 8 months and 27 months of age demonstrated symmetric patchy signal abnormality with T2 hyperintensity throughout the bilateral cerebral white matter indicative of delayed myelination or nonspecific leukoencephalopathy, and mild white matter volume loss with relative thinning of the corpus callosum. Her OFC at the age of 5 years was 46cm (-4 SD). She was hospitalized at 6 years old for septic shock in setting of febrile illness. At the last clinical examination at 7 years of age she presented with progressively worsening mental status alteration, vomiting and constipation. Her weight was 13.6 kg (-5 SD) and height of 103 cm (-4 SD),. No dysmorphic facial features were observed, she was highly hypertonic with athetoid extruded tongue posture. Metabolic screening gave normal results. Molecular genetic work-up included comparative genomic hybridization and Prader-Willi methylation testing with normal results. Trio-WES with DNA samples of both unaffected parents and the proband, revealed a *de novo* heterozygous variant c.2344C>T, p.(Arg782Trp) in *DHX30* (NM_138615.2).

Individual 12 is an 8-year old female, the eldest child of non-consanguineous Australian parents of Caucasian descent. Her siblings and both parents are well and healthy and there is no other family history of note. She was delivered at 40 weeks after an induced rapid and uncomplicated labour. Her birth weight was 3610g (+0.4 SD), her birth length was 49cm (-1.2 SD) and her OFC was around 36-37cm (+0.9 / +1.6SD). She was in good condition at birth and there was no complication in the immediate newborn period. She had feeding problems that have improved but she still chokes on crackers, and she has ongoing constipation issues. Her general health is otherwise good. She has strabismus and cortico-visual deficit, her vision has been improving over time. She has not had any obvious seizures. She has trouble regulating her temperature, she is often cold and wakes up at night when cold. She has inappropriate response to pain (laugh) and she needs strong stimulus (loud noise, strong light) to respond. She smiled at around 12 weeks of age, rolled both ways at 4 months, sat up at 12 months, crawled at 13 months and walked at 4 years of age. Her gait is unsteady and ataxic and she has numerous falls. She has global developmental delay and is non-verbal. She is quite social but doesn’t care to interact with people. She is generally placid. She has a high pain tolerance and is not aware of danger. She holds her hands in a particular position with the thumbs abducted. At her last examination, her growth parameters were all around the 50^th^ percentile. She had deep set eyes (family trait), furnished eyelashes, thin straight eyebrows, strabismus subtle facial asymmetry, posteriorly rotated ears with small antitragus, short antihelix, and everted lower lip; she has joint hypermobility of her hips, shoulders and fingers and A brain MRI showed minor periventricular leukomalacia. Plasma amino acids, urine metabolic screen, carbohydrate deficient transferrin, plasma acid carnitine profile, plasma and CFS amino acid and plasma and CFS pyruvate and lactate were all normal. Chromosome microarray testing detected a small maternally inherited, unlikely significant, 2q12.2q12.3 duplication. Methylation studies for Angelman syndrome did not detect any anomaly. Genetic testing for lysosomal disorders indicated that she is a carrier of Tay-Sachs disease (normal levels of beta-hexosaminidase A activity) and Pompe disease. Genetic testing for an in-house absent speech gene panel did not detect any likely causative variant. Trio-WGS with DNA samples of the proband and both unaffected parents, revealed a *de novo* heterozygous variant c.2344C>T, p.(Arg782Trp) in *DHX30* (NM_138615.2).

Individual 13 is an 8 year old male, the only child of non-consanguineous parents of Mexican origin. Regarding family history, mother has 2 brothers, both of whom had seizures during childhood and have normal cognition. The father has 2 brothers and 1 sisters. Father’s brother's daughter did not talk at the age of 4, walked by age 2 and has a brother who did not talk at the age of 2. Regarding the perinatal history, the pregnancy was uncomplicated and he was born at full term. Birth weight was 3.72 kg. He was healthy in the newborn period except for transient hypoglycemia treated with bottle feeding. It was noted at 6 months that he was microcephalic and had delayed motor milestones. At 6 years of age regarding expressive language development he was able to say mom, dad, and name of dog. He was not able to point or wave. He could do a high five. In regard to gross motor development, he was able to roll, pull to stand, army crawl, and walk with walker. He could crouch down and balance himself. Regarding fine motor skills he was able to do a raking grasp. He could put things in his mouth. He was not toilet trained. He had dysphagia. He could eat finely chopped foods and had difficulty with chewing. In regard to social skills: he was very social. In regard to academics/cognitive ability: he could recognize things that he likes. He was able to identify four colors. At the last comprehensive examination his OFC was 47.5 cm (-2.05 SD), height was 112 cm (-1.77 SD) and weight was 16.2 kg (-2.96 SD). Facial features were elongated. Head shape exhibited microcephaly. Forehead revealed prominent brow. There were epicanthal folds. He had arched eyebrows and long eyelashes. He had temporal muscle wasting. He had a small mouth, down turned. Mental examination revealed a social smile. He was nonverbal. He had a normal cranial nerve examination. He had central and axial hypotonia. He had increased tone in the Achilles. He had normal reflexes. He had joint hypermobility. He was able to walk with an ataxic gait with two hand assist. Diagnostic studies included an MRI of the brain which was normal, one EEG revealed bifrontal and left central parietal sharp waves during sleep but subsequent ones were normal. Hypotonia panel demonstrated normal results for SMA, myotonic dystrophy, Prader Willi, Maternal UPD 14, and Angelman syndrome. Lactate, pyruvate and urine organic acids were normal. Fragile X screen normal. Chromosomal microarray was normal. Cornelia de Lange testing revealed a VUS in the RAD21 gene that was determined to not cause CDLS or any genetic condition. Trio Autism/ID Xpanded Panel with DNA samples of both unaffected parents and the proband, revealed a *de novo* heterozygous variant c.2345 G>A, p.(Arg782Gin) in *DHX30*. (NM_138615.2).

Individual 14 is now a 6-year old male product of a full term normal spontaneous vaginal delivery, the third child of unaffected, non-consanguineous parents of Hispanic and German ancestry. His family history includes two full siblings: an 8-year old brother with speech delay, an 11-year old healthy sister, and two half-siblings through the father: a 10-year old brother with learning disabilities/ADHD and a 13-year old sister with scoliosis. Father is healthy and mother experiences anxiety and depression. At about two months of age, his grandmother noted that he rarely cried and he had significant head lag and was "limp" by her account. His pediatrician formally evaluated him at 6-months of age and requested an ultrasound of his head due to his having a small fontanelle. He received an MRI of the brain as well as an encephalography, which was normal. His Newborn Screen was consistent with sickle cell trait. He also had poor weight gain, acid reflux, and constipation, and was referred to GI, and subsequently gained weight on a high calorie mix. He had high tolerance to pain and reportedly never cried. He was receiving physical therapy and occupational therapy. Developmentally, he began rolling at about 4.5 months, but does not crawl or walk. He presented at the genetics unit at 4-years of age for severe developmental delay and profound generalized hypotonia and dysmorphic features suggestive of a genetic etiology. He could sit up with assistance, but was unable to hold his bottle and had some head control. He could grasp objects and occasionally reached for things. He lacked speech but made some babbling, and cooing, without consonant sounds. His physical features included microcephaly, bitemporal narrowing, a tall forehead, a high arched palate, and a pointed chin with no dimple. Other notable features were a single palmar crease on his left hand, overlapping second and third toes on right foot, hypermobility in his knees, and decreased calf musculature. He had diminished bulk, and was severely hypotonic throughout his body. On vertical suspension, he slipped through the examiner’s hands, and on horizontal suspension was not able to lift his head. He showed a severe lag when pulled from a laying to a sitting position. In a prone position, he had some head control and was able to lift his head occasionally. He could not elicit upper extremity reflexes. He would withdraw upon experiencing light touch. He was unable to sit, even when held in a sitting position. Prior negative testing included a Prader-Willi syndrome methylation assay. Metabolic investigations, including carnitine, lactate/pyruvate, urine organic acids, and plasma amino acids were non-diagnostic. Given his significant hypotonia, testing for Spinal Muscular Atrophy, creatine kinase level, and myotonic dystrophy was pursued; none was informative. Trio-WES with DNA samples of both unaffected parents and the proband, revealed a *de novo* heterozygous variant c.2345 G>A, p.(Arg782Gin) in *DHX30*. (NM_138615.2).

Individual 15 is a 2 year old male, the only child of unaffected, non-consanguineous healthy Dutch Parents of Caucasian European descent. Pregnancy was uncomplicated with normal screening ultrasound. The boy was born at 41 weeks and 6 days. There was meconium in the amniotic fluid. He had APGAR score 4/7/7 after 1, 5 and 10 minutes respectively. Oxygen was supplied shortly and he received antibiotics because of suspected perinatal infection. Birth weight was 3320 grams (-1 SD). Hernia inguinalis was noted. Because of hypotonia, tube feeding was started for the first few days. Psychomotor development was delayed, with laughing at 8 weeks. Physiotherapy was started at age 5 months because of delayed motor milestones with persistent axial hypotonia including head lag. Rolling over at age 9 months. He has gastro-oesophageal reflux and frequent choking when drinking fluids. He experienced recurrent otitis. He never cries and had a high threshold for pain. He has a cheerful behavior. Furthermore, he has a trigger finger. He does not experience seizures and electroencephalogram (EEG) at age 1.5 y was normal. At the last physical examination at age 2 years and 7 months, height is 93 cm (-0.31 SD), weight 14 kg (-0,23 SD), head circumference 47 cm (-1,55 SD). There is a broad and high forehead, low position of the ears, overfolded helix, simple ears, flat midface, brachycephaly, clinodactyly of the 4th and 5th toe, and hypoplastic toe nails of the 5th toe on both sides. He is mildly bradyphrenic and vocalizes but does not speak. There are feeding difficulties. He cannot sit without support, there is still some head lag and axial hypotonia, strabism with saccadic ocular movements.and mild generalized chorea.and poor fine motor skills. ). Metabolic investigations were unremarkable. Brain imaging has not been performed. There was a normal CGG repeat length of the *FMR1* repeat. Trio-WES with DNA samples of both unaffected parents and the proband, revealed a *de novo* heterozygous variant c.2345 G>A, p.(Arg782Gin) in *DHX30*. (NM_138615.2).

Individual 16 is a 4 year female- Family history was unremarkable. She has two older, unaffected half siblings (different fathers). She was delivered at 39 weeks by induction because of absent fetal movements for 2 days, confirmed on ultrasound. Birth weight was 3900 grams (+1.33 SD) and birth length was 48.3 cm (-1.3 SD). She did not cry, but did make grunting noises. As she got older her development fell further behind. She did not cry after vaccinations and she had a "vacant" look and poor eye contact. At 9 months of age she began rolling over, at 11 months she could only sit with support, and she made no attempts to crawl. At the clinical examination at 10 months she had a pronounced global developmental delay. She had significant hypotonia but began showing better eye contact. She was found to have a bicuspid, stenotic aortic valve and a moderately dilated ascending aorta (z score 3.9) and mild insufficiency. She developed staring spells, and a Video EEG showed focal slowing and epileptiform discharges. An MRI showed diffuse subcortical and periventricular white matter signal abnormality in a symmetric pattern involving the temporal frontal and parietal lobes with associated abnormal cerebral volume loss suggestive of a leukodystropy but not typical for periventricular leukomalacia. Organic acids, VLCFA, pipecolic acid and arylsulfatase enzyme testing were normal. Over the next several years, she developed few skills. She said a few words, but none consistently. She had a grand mal seizure age 3, Keppra was started, and she had only one further seizure. At age 4, weight was 15.9 kg (48 %ile) Height: 101.6 cm (52 %ile), She had mild ptosis, hypotonia, and did not talk, stand for very long, or take steps. Because of recurrent ear infections tonsillectomy and adenoidectomy, and tympanostomy tube surgeries were performed at the age of 4. WES revealed a heterozygous variant c.2353 C>T, p.(Arg785Cys) in *DHX30* (NM_138615.2).

Individual 17 is a 7.5-year-old girl, the only child of unaffected, non-consanguineous American parents of Caucasian European descent. Family history was non-contributory. The pregnancy was uncomplicated with normal screening ultrasounds. She was born at 39 weeks of gestation by caesarean section due to breech presentation. Her birth weight was 4054 gram (+1.7 SD), birth length was 53.34 cm (+1SD), and OFC was 38.1 cm (+2.7 SD). Muscular hypotonia was first noted at 8-week old. Since then, she was regularly in physical therapy, and she has had no period of regression. Her milestones of motor development were delayed and has poor fine motor skills. She was able to sit independently at 13 months and to walk without assistance at 37 months of age. She presents symptoms of ataxic gait. She is unable to toilet train, and she was unable to feed herself, but occasionally she can grab food with her fingers to feed herself. She is non-verbal and has been on speech therapy regularly since 1-year-old. Her progress: At one-year-old she could respond to pictures/flashcards. At three-year-old she was able to use a communicator with eye gaze technology. At seven-year-old she was able to transition to a touch screen communication tablet. Overall, she is described as a pleasant, quiet, smiling child with no behavioral concerns. But she is easily irritated by noises. Sleep disturbance is rare. She presents repetitive finger snapping and teeth grinding on a regular basis, which causes trouble in chewing. Physical examination revealed no obvious dysmorphic features or developmental features, except joint hypermobility and low muscle tone, which has improved progressively with physical therapy. Her reflex response findings overall were unremarkable. OFC at last examination at the age of 7years and 8months was 53 cm (+0.9 SD). MRI was performed at 13-month, 17-month, and 37-month-old with similar findings: generalized decreased in white matter volume in a symmetric fashion. The spectroscopy tracing did not reveal any specific abnormality. Ethmoid sinus and left mastoid air cell opacification were noted. She occasionally blanks out or stares into space. Electroencephalogram (EEG) at two-year-old was normal during wake and sleep stages, ruling out absence seizures, and she has not had a history of seizures. She underwent array CGH, Prade-Willi analysis, extensive metabolic analyses (including plasma amino acids, lactate and pyruvate, etc), Canavan disease panel, Krabbe disease test, and lysosomal disease test: all were negative. Trio-WES with DNA samples of both unaffected parents and the proband, revealed a *de novo* heterozygous variant c.2353 C>T, p.(Arg785Cys) in *DHX30* (NM_138615.2).

Individual 18 is a now 3-year-old male. He is the first child of unaffected, non-consanguineous Caucasian parents. There was no family history of seizures, developmental problems, neuromuscular disorders, or other neurologic issues in the family. The pregnancy was uncomplicated aside from polyhydramnios noted in the last month of the pregnancy. He was born via Caesarean section at 40 weeks due to a face presentation. There were no other perinatal or neonatal complications, and he went home from the hospital in a normal amount of time. Developmentally, all milestones were delayed. He did not roll until 6 months, and started to sit up independently at about a year of age. At his last visit at age 3, he could pull to stand and was working on walking with a walker but could not yet walk independently. His fine motor skills were somewhat delayed but he was able to feed himself using a spoon. He had chronic dysphagia and had trouble with chewing and swallowing, though this improved over time. He could copy some sounds but could not yet produce intelligible speech. He could partially communicate needs using a picture board, and does produce some verbal cues that his parents can understand. He was noted to be friendly, social, and interactive with family. He has always made slow, gradual progress and has never regressed. His notable medical problems are strabismus, suspected mild central visual impairment, dysphagia, and failure to thrive. There has never been any evidence of seizures. At his visit at 16 months, he was microcephalic (3%, Z=-1.95) with weight at the 10% for age and height at the 12% for age. Over time, he had had poor growth, and at his last visit at age 3 he was noted to be at the 2nd percentile for height. He also had poor weight gain, with his weight also falling below the 3rd percentile over time, though at the last visit his weight gain had improved after intensive effort by his parents and he was at the 30% for weight. At his last visit at age 3 his head circumference was at the 2nd percentile for age (Z- 2.05) Facial features were nondysmorphic, and he had no notable birthmarks or other malformations. On neurologic exam, he was alert and appropriately attentive to the examiner. He had mild strabismus. He had mild axial and truncal hypotonia and mild symmetric proximal weakness. He could reach for objects without ataxia. Deep tendon reflexes were normal and symmetric, though he had bilateral upgoing toes on Babinski testing. An MRI scan at the age of 1 year showed mild-under operculization of the Sylvian fissures, but was otherwise unremarkable. EEG was not performed. He had an extensive metabolic workup done which was unrevealing including CK, CMP, serum amino acids, urine organic acids, acylcarnitine profile, tsh, carnitine, and pyruvate all of which were normal. He had a normal chromosome microarray. Trio-WES with DNA samples of both unaffected parents and the proband, revealed a *de novo* heterozygous variant c.2353 C>T, p.(Arg785Cys) in *DHX30* (NM_138615.2).

Individual 19 is a 2y8m old female, the second child of unaffected, non-consanguineous parents of Cuban and African-American ancestry on the maternal side, and Irish, Native American and Cape Verdean ancestry on the paternal side. Family history is non-contributory. There were no medical complications during the pregnancy, and she was delivered at full term via induced vaginal delivery due to poor fetal movement. Her mother and father were ages 21 and 20, respectively, when she was born. Phototherapy was needed for hyperbilirubinemia, but otherwise the neonatal course was uncomplicated. She passed the newborn hearing screen, but later had borderline hearing tests and is awaiting brainstem auditory evoked potential test to rule out hearing loss. The patient was first evaluated by neurology at 20 months for concerns of diffuse low muscle tone and global developmental delays. Her neurologic examination was notable for diffuse axial and appendicular hypotonia with preserved deep tendon reflexes. She did not start to combat crawl until 10 months, and started walking at 22 months. MRI of the brain done at 29 months showed no structural abnormalities and normal myelination for age. She has never had any seizures. Clinical examination at 25 months was notable for a weight of 9.895 kg (-1.6 SD), length of 79.9 cm (-2.2 SD), and OFC 47.0 cm (-1.2SD). She had a 5 mm café au lait macule on the right torso and an irregular hypopigmented lesion on the right buttock, and epicanthal folds. Her delays have persisted. She began using SMO braces at 26 months. Her fine motor skills are behind, and she can only stack two blocks, one on top of the other. Beginning around 30 months, autistic features were first noted. Currently, at 2y8m, she has had some language regression and cannot remember words that she had learned earlier. She only uses one word consistently, which is the name of her brother. She has a happy demeanor and does not cry. She has a very high pain tolerance, has bruxism, and stomps her feet repetitively. Although she appears happy, she has a limited repertoire of emotions, makes only intermittent eye contact, and generally appears somewhat indifferent and disinterested regarding interactions with either peers or toys. In addition to this limited social-emotional reciprocity, she also has feeding difficulties, perseverates, and is strong willed.Initial genetic testing included a G-banded karyotype, and chromosomal microarray; both were negative. Trio-WES with DNA samples of both unaffected parents and the proband, revealed a *de novo* heterozygous variant c.2353 C>T, p.(Arg785Cys) in *DHX30* (NM_138615.2).

Individual 20 is a 16-year-old male, the first child of healthy, non-consanguineous parents of European descent. He has a 10-year old-sister with ADHD and dyslexia, but otherwise normal development. A cousin of the father had dyslexia and developmental delay, remaining family history was unremarkable. He was born after an uneventful pregnancy at 39 weeks of gestation with a weight of 3050 g (-1 SD), a length of 54 cm (0.91 SD) and a head circumference of 36 cm (0.54 SD). In the first months of life feeding difficulties and neck asymmetry were reported, and at age 8 months microcephaly was noted. He had psychomotor developmental delay. Age of sitting was at 9-10 months and age of walking at 20-21 months, gait was unstable and ataxic. He is non-verbal. MRI at age 16 months and EEG were normal. Sleeping difficulties slightly improved with Melatonin. He has mild constipation and received tympanostomy tubes. He has a short attention span, is very active and occasional has temper tantrums. At the last physical examination at 16 years and 10 months, his height was 174 cm (-0.62 SD), his weight was 69 kg (0.21 SD), and his OFC was 51.5 cm (-3.39 SD). Facial dymsmorphism included overfolded helices, a low forehead, narrow palpebral fissures, a short philtrum, a high and narrow palate, prominent incisors, a prominent jaw, broad thumbs, gynecomastia and pes valgus. He was friendly but with short attention span, did not speak and showed stereotypic hand movements. Karyotyping, chromosomal microarray analysis, Angelman syndrome methylation testing and sequencing of UBE3A, MECP2, TCF4, CDKL5 and ARX were normal. Trio-WES with DNA samples of both unaffected parents and the proband, revealed a *de novo* heterozygous variant c.2354G>A, p.(Arg785His) in *DHX30* (NM_138615.2). Additionally, a hemizygous, maternally inherited variant of unknown significance c.10688G>A, p.(Gly3563Asp) was identified in *HUWE1* (NM_031407.5).

Individual 21 is a 15 year old female, the first child of unaffected, non-consanguineous parents of Latvian and Norwegian descent. She has a healthy 11 year old brother, and unremarkable family history. The mother experienced hyperemesis and vomiting during the entire pregnancy. The girl was born with elective Caesarean section at gestational week 39. Birth weight and length were 3120 g (-0.5 SD) and 47 cm (-1.9 SD), respectively. She was operated twice, at 3 months and 3 years of age, for inguinal hernias, and had bilateral auricular surgery at age 8 years because of prominent ears with uneven size and unilateral irregular cartilage of the antihelix. Psychomotor development was reportedly normal. Age of sitting was at 6-7 months and age of walking at 12 months, she spoke first words at 11 months. After an unremarkable psychomotor development, a progressive balance impairment with midline ataxia was noted from the age of 8 years. Subsequently, she developed reduced motor skills and cognitive problems with reduced concentration and fatigue. Neurological examination revealed midline and appendicular ataxia with nystagmus on lateral gaze, dysdiadochokinesia, dysmetria and intentional tremor, broad-based ataxic gait and negative Romberg’s test. Metabolic screening was normal. Muscle biopsy histology and testing for mitochondrial respiratory chain defects gave normal results. MRIs showed progressive cerebellar atrophy. Although she had no clinical seizures, EEG showed epileptiform, spike-and-slow-wave activity localized in the left central temporoparietal region. She was given levetiracetam for a period, but this was discontinued following a normal EEG. Audiometry was normal. At the age of 15 years her height was 172cm, her weight was 53kg (-0.3 SD) and her OFC was 55.5 cm (+0.6 SD). Karyotyping with G-banding (46,XX) and array-CGH showed normal results. Trio-WES with DNA samples of both unaffected parents and the proband, revealed a *de novo* heterozygous variant c.2606G>A, p.(Arg908Gln) in *DHX30* (NM_138615.2). Additionally, a *de novo* heterozygous variant of unknown significance c.1535A>C, p.(Glu512Ala) in *KLB* (NM_175737.3) was identified, a gene variants in which have so far not been connected to a human phenotype.

Individual 22 is a 5 year 10 month old female with non-consanguineous parents. She was born after an uncomplicated pregnancy and delivery. Family history is non-contributory. She was born at 41 weeks, birth weight of 4110 g (+1.2 SD), length of 52 cm (-0.1 SD), and OFC of 36 cm (+0.6 SD). Her neonatal course was unremarkable. Her parents became worried when she began to walk around age 1 year because she was unsteady on her feet. She still has an unsteady gait, especially when she is tired. Developmental delay is global and most pronounced for expressive language where her parents estimate that she lags 1-2 years behind her peers. She is starting in ordinary school with extra help. She is a willful child who has some difficulty interacting with other children and often seeks the company of adults. She is very active and needs adult supervision at all times when awake. She wanders at night and ends up in her parents’ bed. Periodically need of melatonin. She has generally been somatically healthy. She had an adenotonsillectomy at age 3 years and has had fewer upper airway infections subsequently. A clinical suspicion of brief complex partial seizures has not been confirmed, she has short non-epileptic abscenses, no AED. Her vision and hearing is normal. She has a bilateral, intermittent, exotropic squint. Ophthalmological exam performed under general anaesthesia at age 3 years was unremarkable. She is neither hypotonic nor dysmorphic. Her height is on the >99 percentile (+2,7 SD), weight 86 perc and OFC 68 perc. Cranial MRI at age 3 years was unremarkable. Array CGH gave normal results. An trio-based, NGS-DDg2p panel revealed a *de novo* heterozygous variant c.347_360del, p.(Ala116Val*fs**12) in *DHX30* (NM_138615.2). No other relevant variants were detected.

Individual 23 is a 3-year-old male, the third child of non-consanguineous Caucasian parents. His brother has a history of visual processing disorder and migraines. His sister has a history of strabismus, ptosis, and delayed walking at 20 months of age. Pregnancy was naturally conceived with pre-existing maternal hypertension treated with amlodipine. Worsening maternal hypertension prompted delivery induction at 37 weeks gestational age. Maternal fever at delivery prompted 24 hours of antibiotics, and after discharge at day of life 3 he required readmission for mild hypothermia. He was noted to have global developmental delay. He started laughing and smiling at 2-4 months of age. He is affectionate with good eye contact with his family, but he does engage in some repetitive behaviors, flaps his arms when excited, and lines objects up. He started sitting at 8 months of age, army crawling at 12 months, pulling to stand at 12 months, and walking at 16-17 months. He had an immature pincer grasp at age 3 years. He did not babble much in early infancy. He said his first word around 18 months. He uses more than 20 words at age 3 years, however his speech is largely not intelligible. Evaluation demonstrated normal growth parameters (height 85th %Ile, weight 40 %ile, head circumference 44 %ile) with hooded eyelids, left eye exotropia, faint synophrys, widely spaced teeth, underdeveloped left pectoralis muscle, and low axial tone. Genetic evaluation included a normal array CGH and fragile X analysis. Trio-WES with DNA samples of both unaffected parents and the proband, revealed a heterozygous variant c.2389C>T (p.Arg797*) in *DHX30* (NM_138615.2) inherited from a mosaic mother. His mother’s history was not notable for any concerns apart from hypertension. His brother was testing and was found not to carry the variant. His sister’s testing remains pending.

Individual 24 is now a 5-year old male, the product of an unremarkable pregnancy and delivery, and the second child of unaffected, non-consanguineous parents of Asian ancestry. He has an unaffected 7-year old brother. At 6-months of age, he was reported to display low muscle tone. His developmental history includes: rolling at 6 months, sitting at 9 months, babbling at 11 months, and walking independently after 12 months of age. After starting to walk, his mother noted he did not progress like his healthy older brother. His first words occurred around 1 year of age, and his vocabulary consisted of 100 words. To this day, he cannot climb stairs. His preference is to run over walking, but he falls frequently. While he has significant language delay, given his sociability with other children, his pediatrician had low concern for autism. He displayed significant drooling though he had adenoid hypertrophy. He first presented at 2 years and 11 months of age to the Neuromuscular Clinic for mild hypotonia, global developmental delay, speech delay, with motor impairment, and no evidence of muscular or peripheral nerve disease. Brain MRI detected T2 hyperintensities consistent with Chiari type 1 malformation with hypomyelination in the parietal occipital lobes; no cerebellar or cerebral atrophy was observed. At three years of age, he last presented to Pediatrics. He was receiving speech, occupational and physical therapies and was reported to have made progress. While reportedly bilingual, upon examination, he did not talk, but he could follow commands, but was not responsive or as communicative as a typically developing child. Physical examination revealed large hyperpigmented patches on his back and buttocks, but lacked cafe au lait spots. He lacked any history of a seizure disorder. Also upon examination, his disposition was smiling and responsive, alert and active. He did not display gross motor defects or localized weakness. His muscle tone was normal, but a little uncoordinated with relaxation. He was able to sit and did not display head lag. He was able to walk unassisted and could rise from the floor unassisted. While he had some decreased facial tone, he had no overt dysmorphic features. At last ascertainment, he was enrolled in Special Education classes. Genetic testing included Fragile X, which was negative, and chromosomal microarray analysis, which detected a likely pathogenic, heterozygous deletion of ~1 Mb at 3p21.31, involving the first 15 exons of *SETD2*, as well as *KIF9*, *KLHL18*, *PTPN23*, *SCAP*, *ELF6*, *CSPG5*, *SMARCC1*, *DHX30* (NM_138615.2) and *MAP4*. Out of these, apart *DHX30* only variants in *SETD2* causing Luscan-Lumish syndrome (OMIM#616831) have been associated with a human phenotype following autosomal dominant inheritance. The literature makes note of altogether seven individuals, with somewhat different phenotype, some of which inherited the *SETD2* variant from unaffected parents. Follow-up testing using DNA samples from both unaffected parents was performed and neither parent carried the deletion, confirming a *de novo* status in the proband.

Individual 25 is a 2 year old male referred for evaluation and management of genetic risk associated with global developmental delay. Family history is positive for maternal anxiety, maternal grandmother with depression and a maternal aunt with autism spectrum disorder who also has an affected daughter. Maternal two brothers with autism spectrum disorder. Pregnancy was complicated by prenatal diagnosis of ventriculomegaly and at birth the patient presented perinatal depression requiring mechanical ventilation. At four months of age the patient showed signs of delayed developmental milestones and he presented seizure activity that responded well to medical treatment. Patient did not sit by himself until 8 months of age, did not crawl, and walked at 18 months of age. He was not verbal until the age of 17 months with a very limited vocabulary by two years of age. He is a happy but hyperkinetic child with a very short attention span for age. On physical examination the patient showed malar hypoplasia, midface hypoplasia, epicanthal folds, almond shaped eyes, stellate pattern of the iris, posteriorly rotated ears, small upturned nose, significant hypotonia and hypermobility. Head MRI confirmed persistent ventriculomegaly with mild reduction of white matter. Next generation sequencing showed an intragenic deletion of exons 7-9 of the *DHX30* (NM_138615.2). *De novo* status was confirmed by Sanger sequencing.
