# Supplementary material for: Genotype–phenotype correlations and novel molecular insights into the DHX30-associated neurodevelopmental disorders
Source: Genome Med. 2021 May 21;13:90. doi: 10.1186/s13073-021-00900-3 (PMC8140440; doi:10.1186/s13073-021-00900-3)
Supplement: Supplementary file 7 — Additional file 7: Figure S4. DHX30 WT acts as an ATP-dependent RNA helicase. [file 13073_2021_900_MOESM7_ESM.docx]

**Additional information for:**

**Genotype–phenotype correlations, and novel molecular insights into the *DHX30*-associated neurodevelopmental disorders**

**Mannucci *et al*.**

**Additional file 7**

**
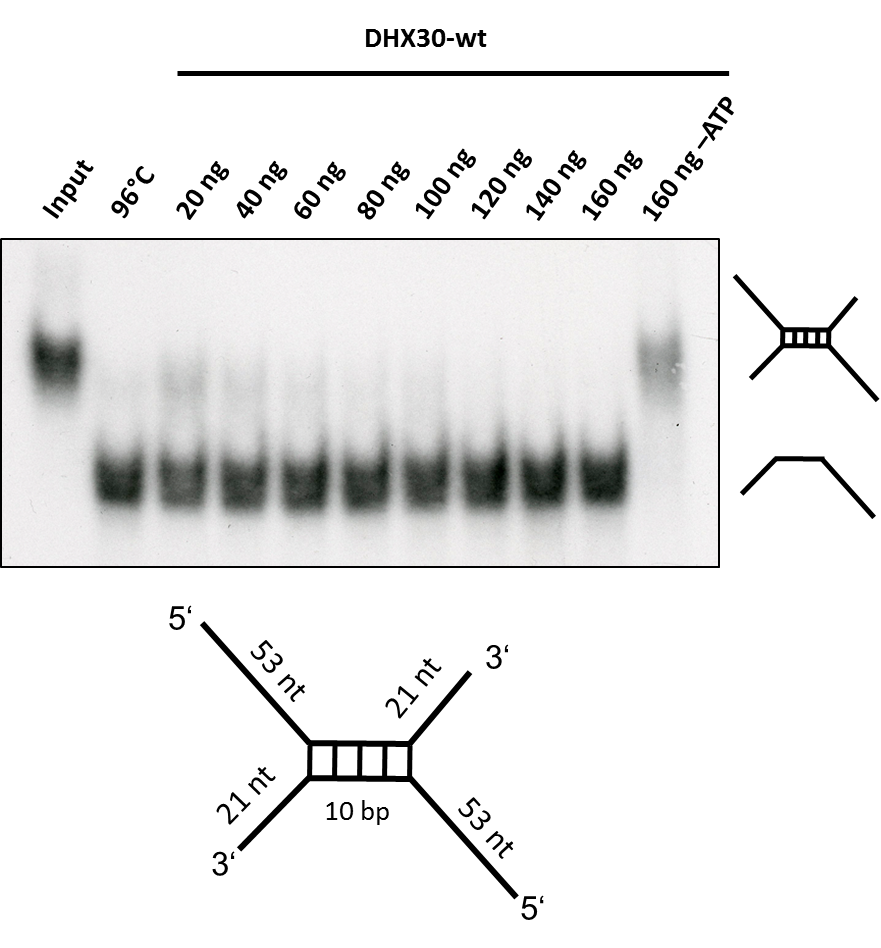
**

**Fig. S4.** **DHX30 WT acts as an ATP-dependent RNA helicase.** Top: Increasing amounts of His6-SUMO-tagged DHX30 WT protein were incubated with a 32P-labelled RNA substrate in the presence (lane 3-7) or absence (lane 8) of ATP and analyzed by native PAGE. The position of the RNA duplex and the single-stranded RNA are indicated in the first and second lane, respectively. Their schematic representation is shown at the right side. Bottom: RNA duplex containing a central GC sequence flanked by single-stranded regions of 53 nucleotides at the 5’ end and 21 nucleotides at the 3’ end.
