# Supplementary material for: Genotype–phenotype correlations and novel molecular insights into the DHX30-associated neurodevelopmental disorders
Source: Genome Med. 2021 May 21;13:90. doi: 10.1186/s13073-021-00900-3 (PMC8140440; doi:10.1186/s13073-021-00900-3)
Supplement: Supplementary file 8 — Additional file 8: Figure S5. Recombinant protein variants of DHX30 induce the formation of cytoplasmic clusters. [file 13073_2021_900_MOESM8_ESM.docx]

**Additional information for:**

**Genotype–phenotype correlations, and novel molecular insights into the *DHX30*-associated neurodevelopmental disorders**

**Mannucci *et al*.**

**Additional file 8**


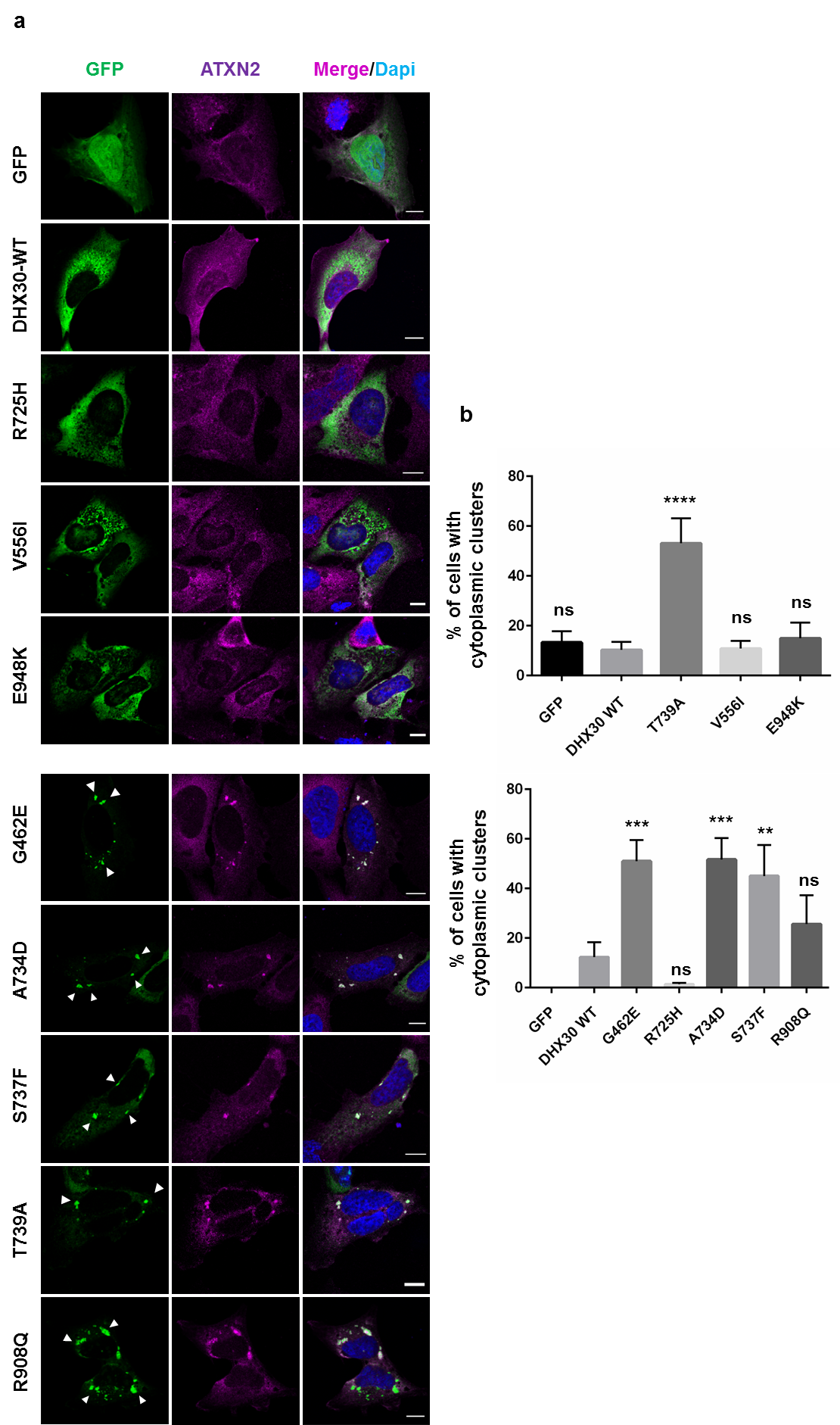


**Fig. S5.** **Recombinant protein variants of DHX30 induce the formation of cytoplasmic clusters.** (**a**) Immunocytochemical detection of DHX30-GFP fusion proteins (GFP, green) and endogenous ATXN2 (magenta) in transfected U2OS cells. Upper panel: wild-type DHX30-GFP preferentially resides throughout the cytoplasm and GFP accumulates in nuclei, similar to recombinant protein variants of DHX30 harboring amino acid substitutions V556I, R725H and E948K (upper panel). Lower panel: recombinant protein variants of DHX30 harboring amino acid substitutions in the helicase core region G462E, A734D, S737F and T739A induce the genesis of cytoplasmic foci containing endogenous SG-marker ATXN2 (arrowheads), Notably, the R908Q amino acid substitution lead to the formation of clusters co-localizing with the SG-marker ATXN2 in only 50% of transfected cells. Nuclei are identified via DAPI staining (blue). Scale bars indicate 10 µm. (**b**) Bar graph indicating the percentage of transfected cells, in which recombinant proteins induce the emergence of clusters. (**,***,****: significantly different form DHX30-WT: **p< 0.01; ***p<0.001; ****p<0.0001; n > 100 from 3 independent transfections; One-Way ANOVA followed by Dunnett’s multiple comparisons test).
