# Supplementary material for: Genotype–phenotype correlations and novel molecular insights into the DHX30-associated neurodevelopmental disorders
Source: Genome Med. 2021 May 21;13:90. doi: 10.1186/s13073-021-00900-3 (PMC8140440; doi:10.1186/s13073-021-00900-3)
Supplement: Supplementary file 9 — Additional file 9: Figure S6. Representative images of zebrafish embryos. [file 13073_2021_900_MOESM9_ESM.docx]

**Additional information for:**

**Genotype–phenotype correlations, and novel molecular insights into the *DHX30*-associated neurodevelopmental disorders**

**Mannucci *et al*.**

**Additional file 9**

**
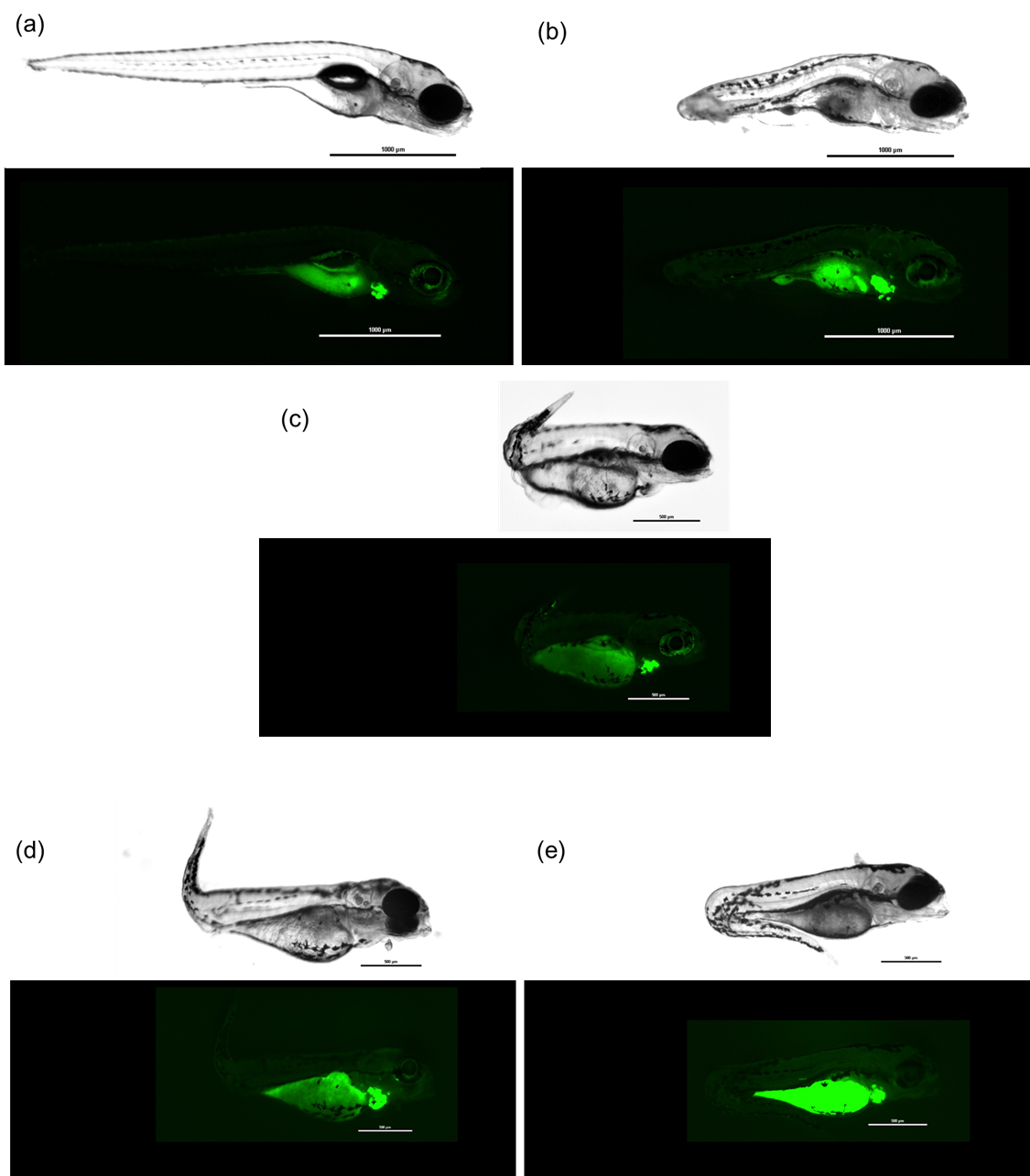
**

**Fig.S6. Representative images of zebrafish embryos.** (**a**) Injected with Tol2 mRNA and pTol2pA2-cmlc2:EGFP;tuba1a:DHX30 wild-type or (**b**) DHX30 harboring R493H, (**c**) R725H, (**d**) R785C, or (**e**) or R908Q. Scale bars show 1000uM or 500 uM per unit. Embryos injected with wild-type DHX30 showed apparently normal development at day 7. Embryos injected with mutated DHX30 showed sign of severe developmental defects before day 7.
