# Supplementary material for: Genotype–phenotype correlations and novel molecular insights into the DHX30-associated neurodevelopmental disorders
Source: Genome Med. 2021 May 21;13:90. doi: 10.1186/s13073-021-00900-3 (PMC8140440; doi:10.1186/s13073-021-00900-3)
Supplement: Supplementary file 10 — Additional file 10: Figure S7. Generation of zebrafish CRISPR-Cas9-mediated dhx30 stable knockout line. [file 13073_2021_900_MOESM10_ESM.docx]

**Additional information for:**

**Genotype–phenotype correlations, and novel molecular insights into the *DHX30*-associated neurodevelopmental disorders**

**Mannucci *et al*.**

**Additional file 10**

**
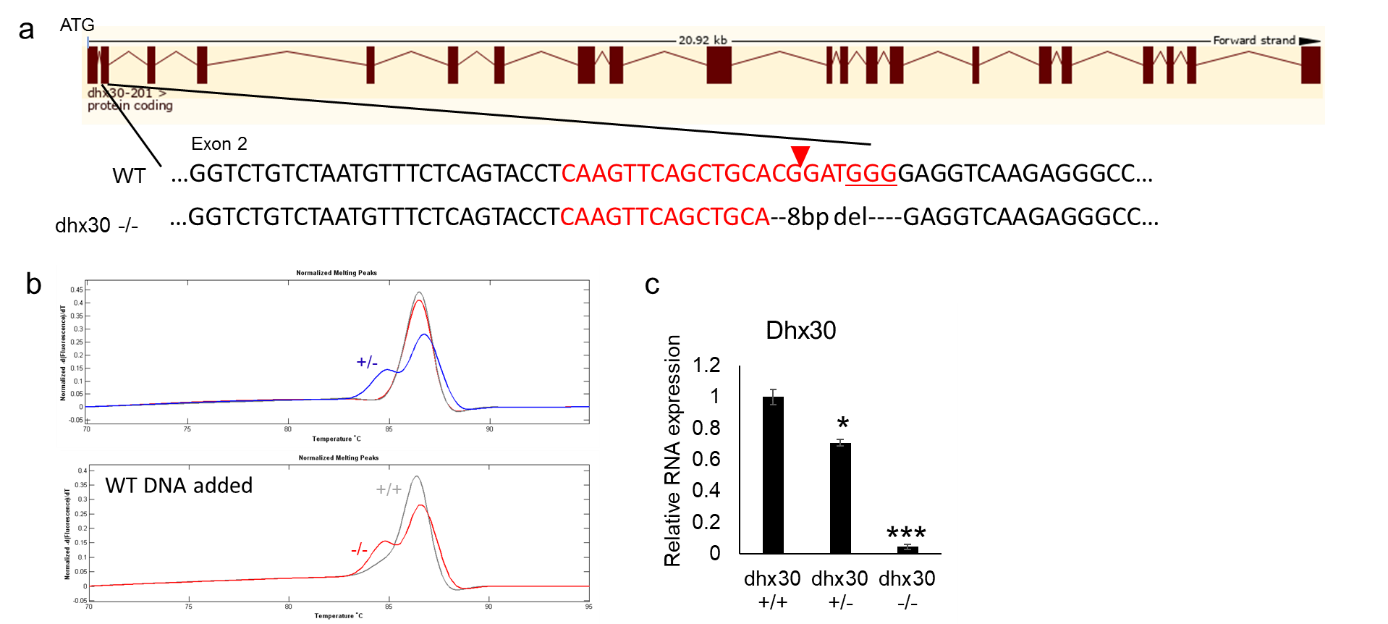
**

**Fig. S7. Generation of zebrafish CRISPR-Cas9-mediated dhx30 stable knockout line. (a)** Shown are genomic regions of zebrafish dhx30 targeted by CRISPR-Cas9. Red indicates gRNA-binding site with the protospacer-adjacent motif (underlined) in wild-type (WT) sequence. The mutant animals carry an 8 bp-deletions generated by CRISPR-Cas9. (**b**) Dhx30-targeted PCR products were analyzed by high-resolution melting analysis (HRM) to distinguish wild-type (+/+), heterozygous (+/-), and homozygous (-/-) animals. Two different melting peaks were shown in heterozygous PCR product (top). To distinguish wild-type and homozygous animals, wild-type DNA samples are mixed with DNA from the ‘test’ animals. Homozygous DNA hence become heterozygous-like, resulting in two melting peaks (bottom). (**c**) Analyses of dhx30 transcript levels in dhx30 mutant animals at 5 days post fertilization. Data are presented as means ± standard error of mean and are based on 3 replications. *, ***: significantly different from DHX30+/+ (*p<0.05, ***p<0.001; n=3; One-way ANOVA, followed by the Holm-Sidak multiple comparison test).
